# Spontaneous Hemopneumothorax: A Rare Cause of Unexplained Hemodynamic Instability in a Young Patient

**DOI:** 10.1155/2020/5026759

**Published:** 2020-01-23

**Authors:** Antonio Higor Marques Aragão, Letícia Aguiar Fonseca, Flávio Clemente Deulefeu, Israel Lopes Medeiros, Rafael Fernandes Viana de Araújo, Carlos Alberto da Cruz Neto, Antero Gomes Neto

**Affiliations:** ^1^College of Medicine, Universidade Estadual do Ceará, Fortaleza, CE, Brazil; ^2^Department of Pulmonology, Hospital de Messejana Dr. Carlos Alberto Studart Gomes, Fortaleza, CE, Brazil; ^3^Department of Thoracic Surgery, Hospital de Messejana Dr. Carlos Alberto Studart Gomes, Fortaleza, CE, Brazil

## Abstract

Spontaneous hemopneumothorax is a rare and potentially life-threatening disorder which complicates about 1-12% of patients presenting with spontaneous pneumothorax and has a remarkable predilection for male patients. It may present with signs of hypovolemic shock without apparent cause. While there are no specific guidelines for the management of patients diagnosed with such condition, wide debate in the literature relating to patient selection for surgery remains unresolved, and recently there seems to be a trend increasingly favorable towards early surgical intervention. Video-assisted thoracic surgery emerges as an excellent option for stable patients and has now been considered the gold standard treatment for spontaneous hemopneumothorax. We report the case of a 17-year-old male patient who presented to the emergency department with a history of sudden chest pain and dyspnea, with no previous evidence of trauma. On admission, the patient presented with hypotension, tachycardia, and cutaneous pallor. Chest X-ray showed hydropneumothorax on the left hemithorax; then, chest tube was placed with an initial drainage of 2000 ml of blood.

## 1. Introduction

Over the past one and a half century, spontaneous hemopneumothorax (SHP) has emerged as a rare well-documented entity in the literature, being first described by Laënnec in 1828, and it complicates about 1-12% of all spontaneous pneumothoraces. Although uncommon, SHP is a potentially life-threatening condition and, therefore, must be remembered in the context of sudden onset of unexplained hemodynamic instability, especially in young males, since proper and immediate management in these cases is a determining factor for both treatment success and to improve prognosis [1].

Although the role of different surgical modalities in the approach to SHP has gained increasing importance over the last few years, the literature still diverges about its indication, the ideal moment for its execution, and the possibility of isolated conservative treatment in selected cases [2–5].

We describe the case of a 17-year-old patient who presented with sudden onset of respiratory distress and signs of hypovolemia and discuss the current state-of-the-art of such condition.

## 2. Case Presentation

**Figure 1:**
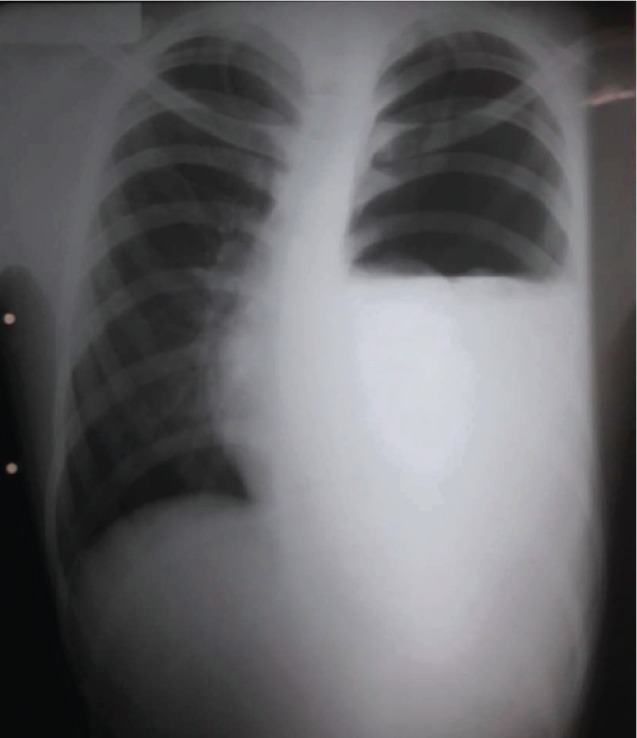
Chest X-ray revealed left pneumothorax with niveau level.

A 17-year-old male patient presents to the emergency department with a two-hour history of sudden onset of left-sided chest pain, exacerbated by breathing, with no radiation to other sites, and associated with shortness of breath. The patient reported no recent trauma and his past medical history was unremarkable. On admission, his vital signs revealed temperature of 34.5°C, blood pressure of 80/40 mmHg, heart rate of 125 beats/min, and respiratory rate of 24 breaths/min with an oxygen saturation of 98% on room air. At physical examination, the patient was dyspneic and presented important painful distress. Cardiopulmonary examination was significant for muffled heart sounds on mitral focus and abolished breathing sounds in the left hemithorax. Electrocardiography (ECG) showed normal sinus rhythm, and initial laboratory results were notable only for a hemoglobin level of 8 mg/dl. Chest X-ray (CXR) evidenced a left-sided hydropneumothorax (Figure 1).

**Figure 2:**
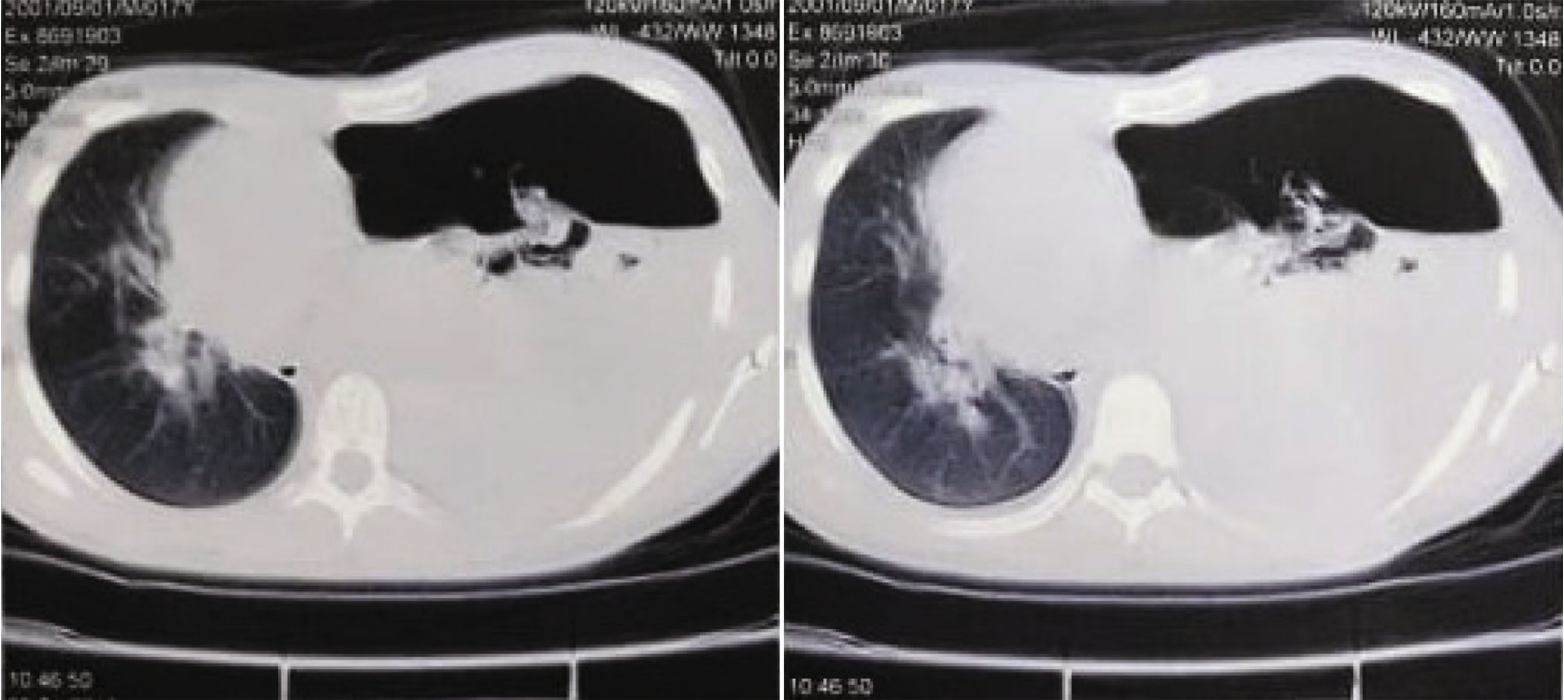
Noncontrast chest CT scan showed hydropneumothorax with left lung collapse and mediastinum deviation.

A computed tomography (CT) scan of the chest revealed left lung collapse with significant deviation of the mediastinum to the right side (Figure 2). A thoracentesis confirmed the presence of blood in the pleural cavity. A chest tube was then inserted in the 5th intercostal space with drainage of air and 2000 ml of blood. Two units of packed red blood cells were administered and the patient's vital signs stabilized as well as his symptoms relieved. The patient was referred to a tertiary cardiopulmonary service for definitive treatment evaluation.

**Figure 3:**
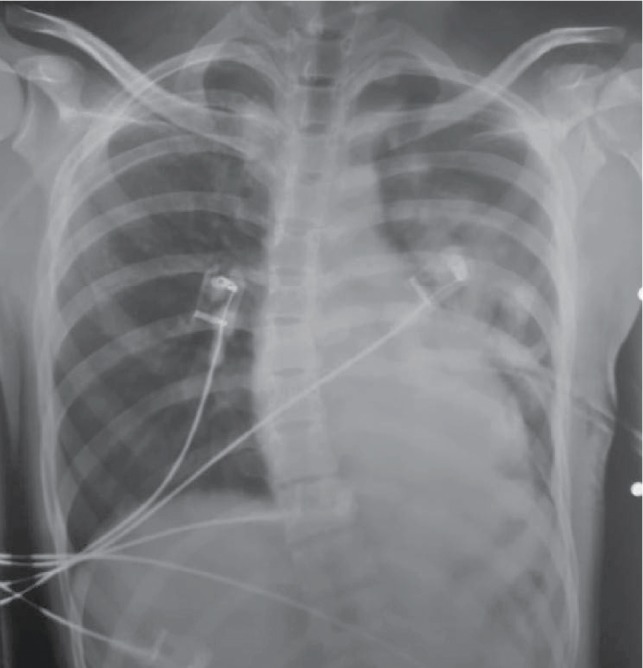
Chest X-ray revealed satisfactory pulmonary expansion after thoracic tube insertion.

Within the next hours till presentation to our service, additional 800 ml of blood were drained. New CXR revealed adequate lung reexpansion after tube insertion (Figure 3), and the thoracic surgery team decided not to proceed to emergency thoracotomy. The patient was admitted, and over the next 24 hours, another 200 ml of blood flowed out from the chest tube. One more unit of packed red blood cells was transfused and signs of hemodynamic instability did not reoccur during hospitalization. Owing to the persistent bleeding, the medical team chose to indicate video-assisted thoracic surgery (VATS) to accomplish definitive hemostasis of the bleeding source.

**Figure 4:**
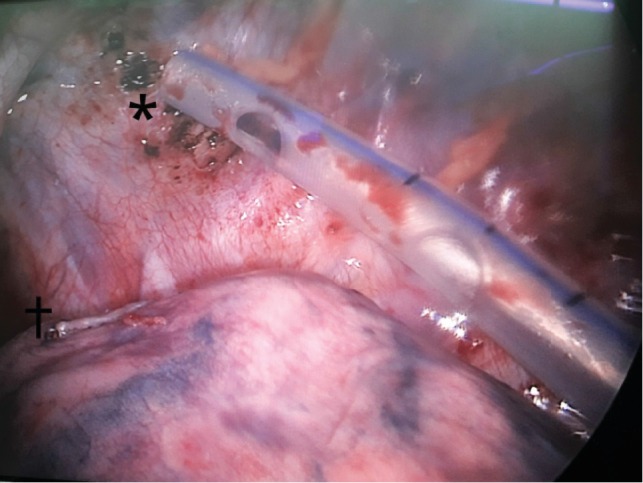
Thoracoscopic visualization of the bleeding source on the top of the left pleural cavity after hemostasis (^∗^) and region of bullectomy (+).

**Figure 5:**
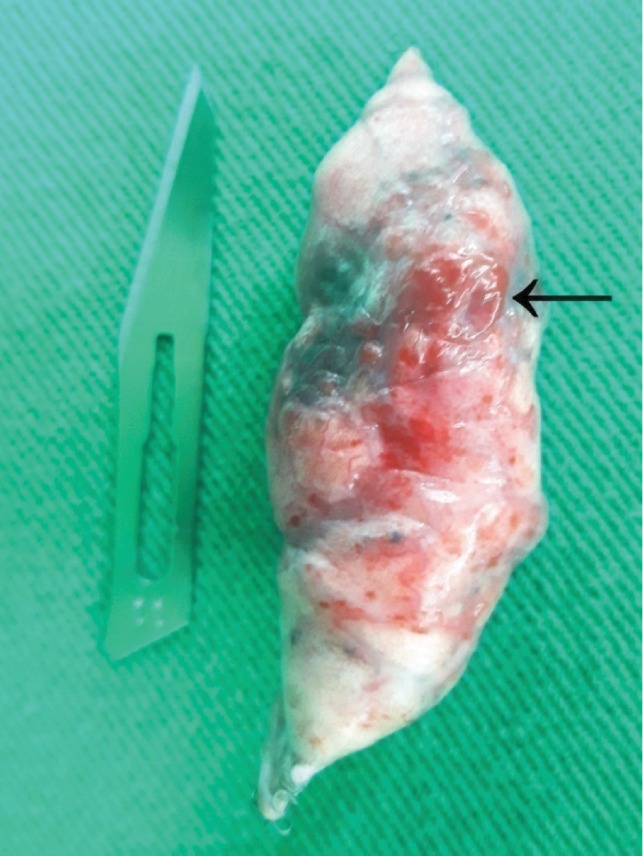
Anatomic specimen operatively resected showing a 0.3 cm diameter bullae in the subpleural space (arrow).

On the third hospitalization day, VATS was performed, after induction of general anesthesia, with camera inserted through the already existing thoracostomy tube incision and confection of a portal in the 4th intercostal space. Exploration of the pleural cavity revealed a large amount of retained blood clot with pleural adhesion exhibiting signs of recent bleeding. A small bullae was found in the apex of the left upper lobe, near the site where a pleural adhesion ruptured (Figures 4 and 5). Complete removal of clots, irrigation of the pleural cavity, electrocauterization of the bleeding adhesion, bullectomy, and abrasive pleurodesis were effectuated. A new port was made in the 7th intercostal space for chest tube insertion and the previous one was closed.

Histopathology showed reactive eosinophilic pleuritis consistent with previous pneumothorax, and no morphological evidence of malignancy or granulomas was present.

The postoperative course was uneventful, and the chest tube was removed on the 7th postoperative day. The patient remained stable and was discharged on the 10th postoperative day.

**Figure 6:**
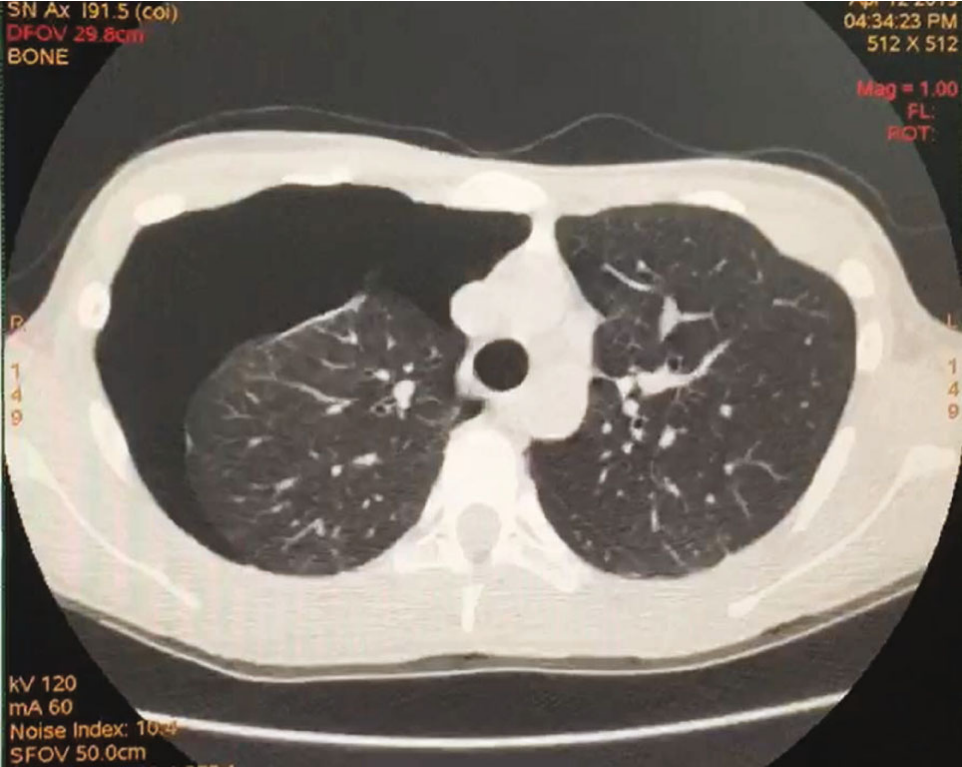
Noncontrast chest CT scan demonstrates the presence of pneumothorax on the right hemithorax.

Seven days after hospital discharge, the patient presents asymptomatic to our service for an outpatient follow-up appointment. However, CXR showed radiologic signs of pneumothorax on the right hemithorax, which was then confirmed by a CT scan of the chest (Figure 6). The patient underwent new thoracoscopy with bullectomy and pleurodesis and was discharged 2 days after the procedure. The patient continued to be reevaluated and no more complications were observed at a 2-month follow-up.

## 3. Discussion

The definition of SHT is not yet unanimous in the literature. Some authors adopt the concept proposed by Ohmori in 1988 as the accumulation of more than 400 ml of blood in the pleural cavity associated with primary spontaneous pneumothorax (PSP) [1, 4, 6]. On the other hand, a recent meta-analysis proposed that any hemothorax accompanying spontaneous pneumothorax is a more reasonable definition for this condition once there are cases reported in the literature in which the initial drainage was less than 400 ml [2, 7].

Almost all described cases of SHT occurred as a complication of a primary spontaneous pneumothorax (PSP). The incidence of SHT has been reported to be around 1-12% of all PSP. A notorious predisposition for male patients is well documented, as the male to female ratio range is approximately 15 : 1, difference significantly higher compared to PSP [1, 7, 8].

The most frequent presenting symptoms are chest pain and sudden dyspnea. Although their initial clinical manifestations may be very similar, the potential development of hypovolemic shock leading to rapid clinical deterioration distinguishes SHT from PSP, as can be observed in our case as well as in many other case reports [6, 9–12]. A study by Kakaris et al. pointed out that, among 71 patients diagnosed with SHT, 29.5% presented hemodynamic instability upon admission [3]. Before this urgency characteristic, we highlight the importance of maintaining a low threshold suspicion for this diagnostic possibility in patients with compatible profile who present with characteristic symptoms.

Upright CXR is a routinely diagnostic tool by demonstrating typical radiological evidence of pneumothorax associated with an air-fluid level, even though up to 10% of cases may present only with pneumothorax on a first moment due to the early execution of this diagnostic testing or to the contention of the bleeding by a pleural adhesion. CT is normally not necessary, but it may be helpful if the diagnosis remains uncertain or to exclude secondary causes of hemothorax [1, 7].

Once diagnosticated, fluid reposition and chest tube insertion must be promptly provided in order to allow pulmonary reexpansion and stabilize the patient. Posteriorly, ultimate treatment decisions must be individualized based on each patient's clinical condition [8, 9].

As surgery indication criteria and the ideal timing for such intervention remain unresolved questions in the literature, there seems to be a trend increasingly favorable towards early surgical intervention in all patients diagnosed with SHT considering the potential for this condition to lead to sudden clinical deterioration and the risks of retained blood clots within the pleural cavity [8, 12].

A retrospective study by Hsu et al. reported that 87.6% out of 201 patients with SHT required surgical intervention after closed tube thoracostomy [4]. In another study by Chang et al., up to 30% of patients initially managed conservatively needed posterior operations or presented prolonged hospital stay owing to later complications such as empyema and persistent air leakage [13]. Surgical strategies for approaching SHT include open thoracotomy or VATS. While the first is the preferred modality on the emergency setting in patients who present with active bleeding and hemodynamic instability, the latter may be performed electively after the clinical condition has stabilized [7].

There are many successful experiences with VATS in the treatment of SHT currently reported [14]. Shorter hospital stay, less blood loss, and decreased need for blood transfusion are mentioned by Chang et al. as advantages experienced by patients who underwent early VATS compared to conservative treatment alone [13]. Moreover, lesser invasive techniques have been refined to optimize these benefits, as attested by a recent report in which a successful uniportal VATS was performed for the management of SHT [10]. In our case, due to persistent blood drainage by chest tube, the patient underwent elective VATS with abrasive pleurodesis and bullectomy, obtaining satisfactory results and fast recovery.

On the other hand, some authors defend that conservative work-up may be sufficient in selected cases. Haciibrahimoglu et al. advocate that thoracic drainage alone is sufficient in most cases, and surgical intervention is expendable if the blood outflow from the drainage tube has stopped in less than 24 hours [5]. A study by Kakaris et al. conducted only conservative treatment in 16 out of 71 patients with SHT [3].

The occurrence of contralateral pneumothorax following an SHT episode is not commonly observed in the literature, as we can see in a review that analyzed 8 case series, containing 201 patients with SHT, in which no recurrence of pneumothorax was observed in a follow-up period that ranged from 5 months to 8 years [4]. On a prospective study by Kim et al., 17 patients were diagnosed with SHT, of which 2 of them evolved with contralateral spontaneous pneumothrax after being initially treated with thoracostomy [8]. Kakamad et al. (2016) reports the case of a patient with SHT who was initially treated with thoracotomy, and after one month was readmitted with contralateral pneumothorax [9]. Our patient presented asymptomatic right pneumothorax one week after being submitted to VATS. To our knowledge, this time interval of recurrence was the earliest among the cases described in the literature.

## 4. Conclusion

Therefore, clinicians acting on emergency departments must be aware of SHT as a diagnostic possibility in young male patients presenting with abrupt onset of hypovolemia signs with no apparent cause, despite being an unusual condition [1].

Currently, predominant opinion supports that all cases of STH should undergo early surgical intervention, but such belief is mostly based on different center case series. Thus, we conclude that additional prospective, randomized, and multicentric trials are warranted to define consistent eligibility or exclusion criteria for conservative treatment as well as to design standardized management algorithms for this entity.
